# Tissue-Specific Differences in Fatty Acid Content and Desaturase Activity Between the Liver and Spinal Cord of Diabetic ZDF Rats

**DOI:** 10.3390/biology14091205

**Published:** 2025-09-06

**Authors:** Katarína Orešanská, Mária Chomová, Janka Kubincová, Ladislav Turecký, Monika Ďurfinová

**Affiliations:** 1 Institute of Medical Chemistry, Biochemistry and Clinical Biochemistry, Faculty of Medicine, Comenius University, Sasinkova 2, 81108 Bratislava, Slovakia; oresanska2@uniba.sk (K.O.); maria.chomova@fmed.uniba.sk (M.C.); turecky1@uniba.sk (L.T.); 2 Food Research Institute, National Agricultural and Food Centre, Priemyselná 4, 82475 Bratislava, Slovakia; janka.kubincova@nppc.sk

**Keywords:** type 2 diabetes, desaturase activity, lipid metabolism, spinal cord, liver, PUFA, neuropathy, animal model

## Abstract

Imbalance in fatty acid (FA) metabolism is a critical factor in the development of type 2 diabetes (T2D). This study examined fatty acid composition and desaturase activities in the liver and spinal cord of male Zucker diabetic fatty (ZDF) rats, a genetic model of T2D. Heterozygous lean ZDF fa/+ animals served as controls, while homozygous obese ZDF fa/fa animals represented the diabetic group. FA profiles were determined by gas chromatography, and the activities of Δ5-desaturase (FADS1), Δ6-desaturase (FADS2), Δ9-desaturase (SCD1), and elongase of very long-chain fatty acids (ELOVL) were estimated. T2D rats displayed significantly elevated levels of monounsaturated fatty acids (MUFAs) and increased SCD1 activity in both the liver and spinal cord. In contrast, polyunsaturated fatty acids (PUFAs), particularly arachidonic acid (AA, C20:4 n-6), were reduced. Since AA plays a fundamental role in neuronal membrane structure and signaling pathways, these alterations have particular relevance to nervous system function. Tissue-specific alterations further suggested impaired FADS1 activity in the liver and reduced elongase/FADS2 activity in the spinal cord. These findings suggest that desaturase imbalance and FA remodeling in the spinal cord might represent characteristic features of T2D and that altered FA metabolism within the nervous system may potentially serve as an early indicator of neuropathy or a predictor of increased susceptibility to diabetes-related complications.

## Simple Summary

Type 2 diabetes (T2D) is a chronic disease that often leads to nerve damage, causing painful neuropathy and serious health complications. Understanding the biological changes that contribute to nerve injury is important for developing better treatments. In this study, we identified alterations in fatty acid composition and in the activities of desaturases in the spinal cord of diabetic rats. These alterations were compared with the changes in the liver of T2D rats. We used a well-established animal model of type 2 diabetes and analyzed the composition of fatty acids in the tissues. Our results indicate that type 2 diabetes induces alterations in lipid metabolism not only in adipose tissue and the liver but also in the spinal cord, potentially contributing to neural damage. This research pointed to possible changes in lipid metabolism associated with hyperglycemia and insulin resistance also in spinal cord tissue. The changes we found could, after detailed analyses, help improve our understanding of diabetic neuropathy and may support future development of therapies to prevent or reduce neural inflammatory diseases in diabetic patients.

## 1. Introduction

### 1.1. Lipotoxicity and Desaturases in Metabolic Regulation

Type 2 diabetes (T2D) is strongly associated with obesity, defined as excessive fat accumulation [1]. When fat (both exogenous and endogenous) exceeds the storage capacity of adipose tissue, it accumulates in ectopic sites (e.g., the heart, skeletal muscle, liver, or pancreas), thereby contributing to the expansion of visceral fat deposits and resulting in free fatty acid (FFA)-induced toxicity, also known as lipotoxicity [2]. Saturated fatty acids in the tissues are oxidized, used for the synthesis of FA-derived metabolites, or modified by elongation or desaturation. Desaturases operate as microsomal enzyme complexes composed of the desaturase itself, cytochrome *b*5, and NADH-cytochrome *b*5 reductase. Stearoyl-CoA desaturase-1 (SCD1; Δ9-desaturase) is the rate-limiting enzyme that introduces a double bond at the Δ9 position of saturated fatty acyl-CoAs, primarily converting stearic acid (C18:0) to oleic acid (C18:1; n-9) and palmitic acid (C16:0) to palmitoleic acid (C16:1; n-7). These reactions represent the major endogenous source of monounsaturated fatty acids (MUFAs) in mammalian tissues. MUFAs play indispensable roles in lipid metabolism by regulating membrane fluidity, serving as substrates for triglyceride and phospholipid synthesis, and influencing signaling pathways [3,4]. However, excessive SCD1 activity leads to an abnormal increase in intracellular MUFA pools, which facilitates the synthesis and accumulation of bioactive lipid intermediates such as diacylglycerols, acyl-CoA, and ceramides. These metabolites are known to interfere with insulin receptor signaling, thereby promoting insulin resistance and dysregulated glucose homeostasis [5,6]. By controlling the balance between saturated and monounsaturated fatty acids, SCD1 acts as a central node in the regulation of intracellular lipid homeostasis and glycolipid metabolism. Dysregulation of this Δ9-desaturase–MUFA axis has been implicated in the development of metabolic dysfunction-associated fatty liver disease (MAFLD), which frequently coexists with T2D [7]. Elucidating the signaling networks governed by SCD1 under both physiological and pathological conditions is therefore essential for understanding its role in energy balance and the pathogenesis of metabolic diseases.

### 1.2. PUFA Metabolism and Insulin Regulation

The fatty acid desaturase-1 (FADS1; Δ5-desaturase) and fatty acid desaturase-2 (FADS2; Δ6-desaturase) are enzymes involved in the metabolism of n-3 and n-6 polyunsaturated fatty acids (PUFAs), enabling the formation of long-chain metabolites from dietary α-linolenic (ALA, C18:3 n-3) and linoleic (LA, C18:2 n-6) acids. Linoleic acid (LA, n-6) is metabolized to *γ*-linolenic acid (GLA, C18:3 n-6) by FADS2 and serves as an important constituent of membrane phospholipids as well as a substrate for prostaglandin synthesis, which is also important for maintaining blood flow. Arachidonic acid (AA, 20:4 n-6), produced via elongation of GLA and subsequent desaturation by FADS1, serves as a substrate for the synthesis of eicosanoids [8]. AA-derived metabolites play crucial roles in chronic inflammation, cardiovascular diseases (CVDs), and cancer [9,10].

**Figure 1:**
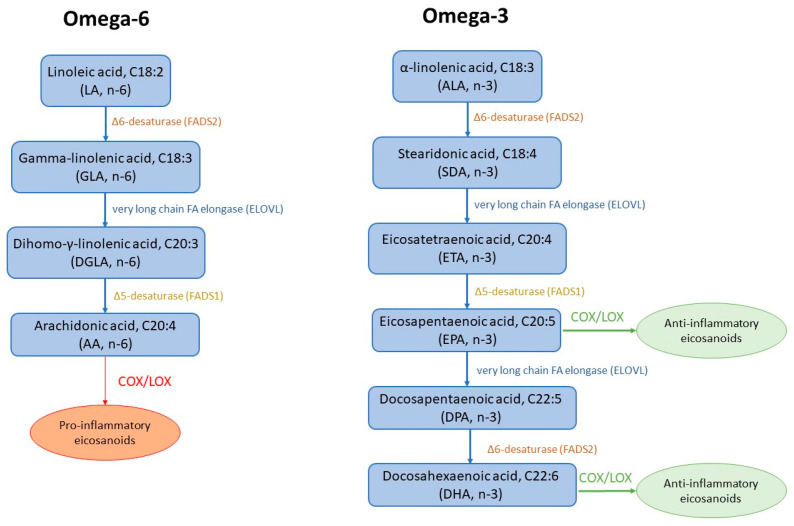
Biosynthetic pathways and eicosanoid derivates of omega-6 and omega-3 polyunsaturated fatty acids. FADS1—fatty acid desaturase-1 (Δ5-desaturase); FADS2—fatty acid desaturase-2 (Δ6-desaturase); COX—cyclooxygenase; LOX—lipoxygenase.

The synthesis of arachidonic (AA) and docosahexaenoic acids (DHA, C22:6 n-3) can be schematically represented in Figure 1.

Arachidonic acid (AA) serves not only as a structural component of membranes but also as a precursor of neuromodulators, including endocannabinoids, which are enzymatically generated from membrane phospholipid-derived fatty acids (FAs) such as AA and DHA in an on-demand manner [11]. The endocannabinoid system acts as a critical homeostatic regulator. It regulates physiological and pathophysiological processes, including neurodevelopment, synaptic transmission, learning and memory, emotional regulation, immunomodulation, hormone secretion, appetite control, and energy balance [12,13].

In parallel, α-linolenic acid (ALA, C18:3; n-3) is metabolized to eicosapentaenoic acid (EPA, 20:5; n-3) and docosahexaenoic acid (DHA, 22:6; n-3), both of which are essential for the synthesis of anti-inflammatory lipid mediators. The biosynthesis of these long-chain polyunsaturated fatty acids (LC-PUFAs) is primarily driven by the FADS1 and FADS2, enzymes tightly regulated by dietary and hormonal factors. Among hormones, insulin uniquely activates both desaturases, and in experimental type 1 diabetes (T1D), suppressed FADS2 activity could be restored via insulin-mediated stimulation of its mRNA expression [14]. Diabetes-associated downregulation is consistently linked to reduced AA content and elevated linoleic acid levels in most tissues, with the brain being a notable exception [14].

The aim of our study was to compare fatty acid profiles and desaturase activities in the liver and spinal cord of Zucker diabetic fatty (ZDF) rats, an experimental model of type 2 diabetes (T2D), in order to determine whether alterations in neural tissue reflects similar metabolic trends to those observed in the liver. Given that the spinal cord has been far less investigated in this context, our study specifically aimed to identify possible tissue-specific lipid remodeling and to explore potential indicators that might serve as early markers of neuropathy in T2D.

Understanding the physiological mechanisms that contribute to specific lipid imbalance in neural tissue may help identify the pathomechanism of neurolipotoxicity or potential early predictive markers for neuropathy risk.

## 2. Materials and Methods

### 2.1. Ethics

All animals were housed at the Department of Toxicology and Laboratory Animal Breeding, Centre of Experimental Medicine, Slovak Academy of Sciences, Dobra Voda, Slovak Republic. The study was approved by the Department of Animal Wellness, State Veterinary and Food Administration of Slovakia (approval Ro-493/18-221/3), in accordance with Directive 2010/63/EU.

All experiments were designed and reported in accordance with the ARRIVE guidelines for animal research to ensure transparent and comprehensive reporting.

### 2.2. Experimental Model

The initial study design included 32 male Zucker diabetic fatty (ZDF) rats, which carry an autosomal recessive missense mutation (fa/) in the leptin receptor gene (Lepr). The control group (CONT) was composed of lean phenotype ZDF heterozygous fa/+ (rats carrying a single copy of the leptin receptor gene mutation). The T2D group was composed of ZDF homozygous fa/fa genotype (two copies of the leptin receptor gene mutation). The homozygous fa/fa mutation results in hyperphagia and subsequent obesity. ZDF rats additionally develop hyperinsulinemia, hyperlipidemia, and impaired glucose tolerance [15,16,17].

This study included 15 male ZDF lean rats (fa/+) in the control group (CONT) and 16 male ZDF (fa/fa) rats. Due to the death of 1 control animal, the final number of rats analyzed was 15 in the control group, and ZDF (fa/fa) rats were divided into two subgroups. In the first group (T2D), diabetes was developed in 8 animals as they fulfilled the predefined criteria for T2D (fasting glycemia > 12 mmol/L). In the second group (obese) (n = 8) were rats with fasting normoglycemia (fasting glycemia less than 12 mmol/L) and hyperinsulinemia. These ZDF obese (fa/fa) rats with normoglycemia were not included in our study as spinal cord samples were obtained only from 4 animals. In our experiments, 23 rats were used as follows: control group n = 15 and diabetic group (T2D) n = 8.

During the experiment, all rats were housed under a 12 h light/12 h dark cycle, at a constant temperature (20–22 °C), with water and food ad libitum. From the 3rd to the 7th week of life, they received a standard pellet diet. From the 8th week onwards, they received Purina Rodent LabDiet 5008 (23.5% protein, 6.5% fat, 3.8% fiber, and 6.8% ash). ZDF (fa/fa) rats typically develop diabetes by 12 weeks of age when maintained on a Purina 5008 diet. Glucose and insulin levels were measured in all rats at 36 weeks of age following a 12 h fasting period. Blood samples were collected from the tail under sevoflurane anesthesia. Blood glucose was assessed using a glucometer (FreeStyle Optium, Abbott, Maidenhead, UK), and plasma insulin concentrations were determined with the Rat/Mouse Insulin ELISA Kit (EZRMI-13K, Merck-Millipore, Darmstadt, Germany).

At the termination of the experiment (38–39 week of life), rats were anesthetized in an induction chamber with sevoflurane (2.5% inspired fraction) until loss of righting reflex. Subsequently, euthanasia was performed by decapitation. Spinal cord and liver tissue were rapidly excised, immediately frozen, and stored at −80 °C until further analysis.

A detailed description of the experimental design, including the genotypes of the rats, as well as changes of the body weight, non-fasting blood glucose, and plasma insulin concentrations throughout the experiment have been reported in [17].

### 2.3. Determination of Fatty Acids by Gas Chromatography

#### 2.3.1. Methylation and Transesterification of the Samples

For the determination of free fatty acid (FFA) content, samples were prepared as follows: 50 mg of each tissue (spinal cord and liver) was weighed and subsequently lyophilized. To the lyophilized material, 1 mL of a saturated diazomethane solution in hexane was added to methylate the free fatty acids. Free fatty acids (C14:0 to C24:1) were extracted and derivatized by methylation. The reaction mixture was incubated at room temperature for 24 h.

The first aliquot was analyzed directly by gas chromatography with flame ionization detection (GC–FID) to quantify FFAs (C14:0–C24:1) as their methyl esters.

The second aliquot was subjected to transesterification to determine esterified fatty acids contained in triglycerides and phospholipids. Fifty µL of sodium methoxide in dry methanol (1:1 dilution of a saturated solution) was added, and vials were shaken for 15 min at 45 °C. The mixture was neutralized with 30 µL oxalic acid in ether, and the sodium oxalate precipitate was removed by centrifugation. To suppress hydrolysis, 20 µL methyl acetate was added; in case of hydrolysis, methyl acetate decomposed preferentially. The resulting solution was analyzed by GC–FID, where esterified fatty acids were detected as methyl esters, while FFAs remained unreacted and eluted at distinct retention times.

#### 2.3.2. Fatty Acid Measurement

Fatty acid analysis was performed using a DB-23 capillary column (Agilent J&W, 60 m × 0.25 mm, 0.25 µm film thickness). Temperature program: initial 60 °C for 1 min; ramp at 25 °C/min to 195 °C; 5 °C/min to 245 °C; and isothermal hold at 245 °C for 15 min.

GC–FID was selected as a validated, highly sensitive, and reproducible method for the separation and quantification of fatty acid methyl esters (FAMEs) in complex biological matrices and remains the method of choice in lipidomic analyses for accurate profiling of individual fatty acids in tissues [18].

A total of 21 fatty acids were identified. Quantification was based on peak area integration and expressed as percentage of total determined fatty acids (% relative abundance). Desaturase activities were estimated by product-to-precursor ratios according to [19,20]: Δ9-desaturase (C16:1/C16:0 and C18:1/C18:0) and Δ5-desaturase (C20:4n-6/C20:3n-6). Δ6-desaturase and elongase activities (C20:3n-6/C18:3n-6) were not assessed separately, as C18:3 was below the detection limit in the spinal cord. Additional fatty acids (C14:0, C18:1t, C18:3n-3, C20:0, C20:1, C20:2, C20:3n-3, C22:0, C22:1, C22:2, C22:6, and C24:1) were included in the total sums of saturated (SFAs), monounsaturated (MUFAs), or polyunsaturated fatty acids (PUFAs) but not reported individually due to their contribution < 2%.

### 2.4. Statistical Analysis

**Figure 2:**
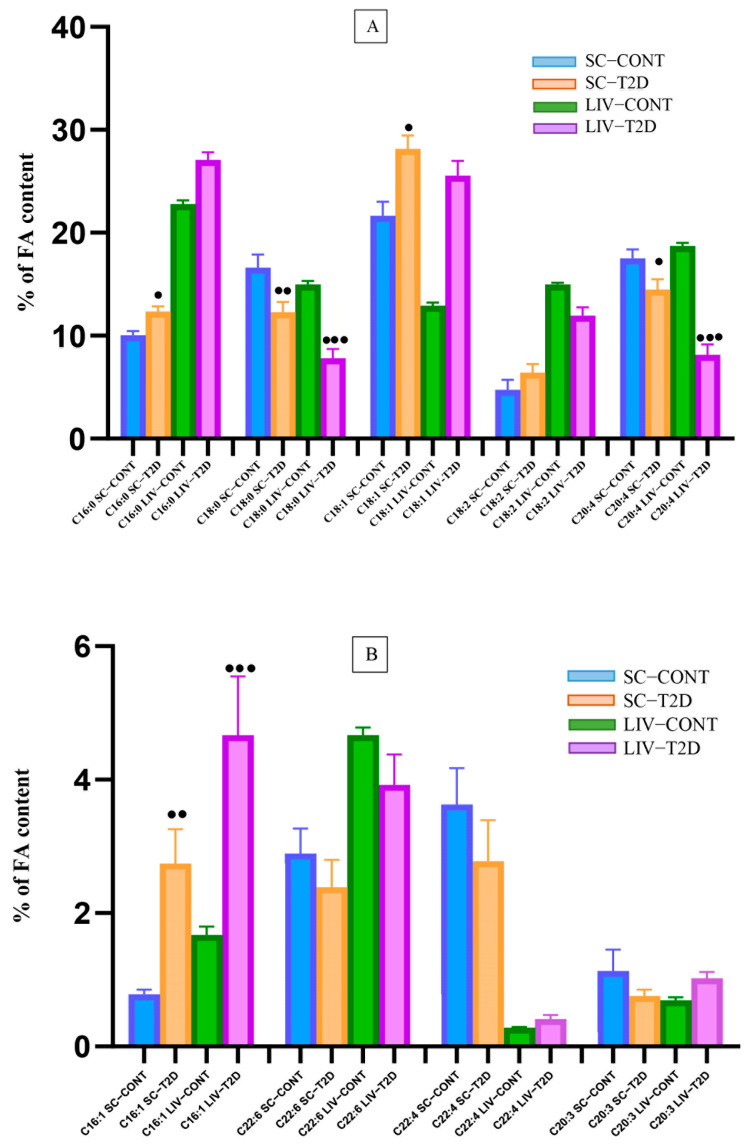
Individual saturated and unsaturated fatty acid content in the liver and spinal cord. Abbreviations: SC—spinal cord; LIV—liver; CONT—control group (ZDF fa/+); T2D—hyperglycemic ZDF fa/fa group. Graph (**A**) shows % content (10–30%) of FAs C16:0, C18:0, C18:2, and C20:4 out of the total determined FFAs (100%). Graph (**B**) shows % content (0–6%) of FAs C16:1, C22:6, C22:4, and C20:3 out of the total determined FFAs (100%). Data were analyzed using ANOVA followed by Bonferroni post hoc test. Comparisons were made between CONT and T2D within the spinal cord group for each FA separately and between CONT and T2D within the liver group for each FA separately. Data presented as mean ± SEM; *p* < 0.05 significance between type 2 diabetes (T2D) and control group (CONT); ^●^ *p* < 0.05; ^●●^ *p* < 0.01; ^●●●^ *p* < 0.0001.

Data are presented as mean ± standard deviation (SD) or mean ± SEM (presented in Figure 2). Grubbs’ test was applied to detect outliers, which were excluded from further analyses. The Shapiro–Wilk test was used to assess the normality of data distribution, and Levene’s test was applied to evaluate the homogeneity of variances. For parametric data, a *t*-test was performed. For non-parametric data, the Mann–Whitney test was performed. Spearman’s correlation analysis was used to evaluate the correlations between parameters.

Group comparisons were conducted by analysis of variance (ANOVA), followed by Bonferroni post hoc testing. A *p* value < 0.05 was considered statistically significant. Statistical analyses were performed using GraphPad Prism 8.0.1.

## 3. Results

Body weight, blood glucose, and plasma insulin concentrations of male ZDF rats are summarized in Table 1.

In the 36th week, T2D rats exhibited significantly higher glucose concentrations compared to control animals (CONT). T2D rats also showed significantly elevated insulin concentrations and higher body weight than control rats.

This may result from a non-significant increase in C16:0 and a significant decrease in C18:0 in both tissues. A significant difference in the percentage of monounsaturated fatty acids (MUFAs) was observed in the tissues of diabetic animals (a 1.7-fold increase in the liver and a 1.2-fold increase in the spinal cord) compared to controls (Table 2). Significantly higher levels of C16:1 (Figure 2B; C16:1 SC-T2D; C16:1 LIV-T2D) were found in both tissues.

In T2D rats, a significant reduction in the percentage of polyunsaturated fatty acids (PUFAs) was observed (1.3-fold lower in the liver and 1.13-fold lower in the spinal cord). In the liver, arachidonic acid (C20:4) was significantly reduced, accompanied by a decrease in n-6 fatty acids and an increased n-3/n-6 ratio (Table 2 and Figure 2A; C20:4 LIV-T2D). In the spinal cord of T2D rats, a significantly lower content of C20:4 and a non-significant increase in C18:2 were observed (Figure 2A; C20:4 SC-T2D; C18:2 SC-T2D). The n-3/n-6 ratio was reduced (non-significantly) in the experimental group compared to the control group. The proportions of other measured PUFAs were comparable between the two experimental groups.

Changes in the percentage content of free fatty acids in tissues observed in the T2D model were not reflected in the total fatty acid content (free + bound), as the total contents of SFAs, MUFAs, and PUFAs did not differ significantly between control and T2D rats.Desaturase activities were expressed as the product-to-substrate ratio (Table 3).

In the liver of T2D animals, increased activity of SCD1 was observed, as indicated by an increase in the C16:1/C16:0 ratio from 0.07 (control) to 0.13 (T2D) and in the C18:1/C18:0 ratio from 0.86 to 2.8. Conversely, a significant decrease in FADS1 activity was recorded (the C20:4/C20:3 ratio decreased from 26.6 in controls to 8.1 in T2D), indicating impaired synthesis of arachidonic acid from dihomo-γ-linolenic acid (DGLA; 20:3 n-6), which is an omega-6 polyunsaturated fatty acid and a metabolic precursor of anti-inflammatory eicosanoids such as prostaglandin E1.

In the spinal cord, SCD1 activity also increased (with the C16:1/C16:0 ratio rising from 0.07 to 0.2). However, the C18:1/C18:0 increase was less pronounced (1.3 to 2.2). The increase in FADS1 activity was noted in this tissue. Since the spinal cord tissue of T2D rats exhibited higher levels of C18:2 and lower levels of C20:3 and C20:4, this may indicate that elongase and FADS2 activities, rather than FADS1, are more affected. This is further supported by a reduced C20:4/C18:2 ratio in T2D rats compared to controls (Table 3).

## 4. Discussion

Type 2 diabetes (T2D) is associated with lipid accumulation in various tissues, including adipose tissue, liver, pancreas, myocardium, and skeletal muscle. Our study focused on changes in fatty acid composition in spinal cord tissue and compared them with those in the liver of ZDF rats. The aim of our work was to assess lipid homeostasis in the spinal cord under conditions of spontaneously developed type 2 diabetes, a relatively underexplored area.

Our study revealed significant alterations in MUFA and PUFA content in both the spinal cord and liver of rats with spontaneous T2D. Our results, consistent with data in the literature, suggest that type 2 diabetes and insulin resistance are linked to altered lipid metabolism, including the spinal cord [21]. This is reflected in altered desaturase activity in the T2D experimental model, also in the spinal cord.

The changes in the FA spectrum in the tissues, together with increased SFA levels in plasma [22], suggest plasma FAs as a major source for tissues, subsequently processed by tissue-specific mechanisms. Increased FFAs in the tissues under T2D (insulin resistance) could, at a theoretical level, be explained by several mechanisms: (1) increased uptake of plasma FFAs into tissues, (2) enhanced synthesis of FFAs directly within the tissue, (3) decreased FFA oxidation, and others. The increased rates of fatty acid transport in the heart and skeletal muscle of obese Zucker rats have been observed and attributed to enhanced translocation of FAT/CD36 transporters to the plasma membrane [23,24]. Based on these findings, and on our observation of increased MUFA percentages in spinal cord and liver tissues, we hypothesize an increased influx of MUFAs into these tissues of diabetic Zucker rats as one of the potential sources of FFAs. Another possible hypothesis is an increased influx of SFAs from plasma into tissues, since storage triglycerides also contain SFAs. Subsequently, these might undergo more extensive desaturation to MUFAs in the tissues of T2D animals. This assumption could be supported by the increased activity of SCD1 in tissues of T2D rats compared with controls. In the liver, conversion of stearic acid (C18:0) to oleic acid (C18:1) was markedly elevated in T2D rats compared with the control group (a threefold increase in the C18:1/C18:0 ratio). In the spinal cord, desaturation of palmitic acid (C16:0) was more pronounced (a twofold increase in C16:1/C16:0). T2D rats exhibited significantly higher C16:0 and lower C18:0 content in both tissues, despite desaturase activity being present only indirectly via product-to-substrate ratios, rather than mRNA or protein expression. Our results support the hypothesis that type 2 diabetes is associated with SCD1 overexpression and overactivity. The finding of increased SCD1 activity in the spinal cord highlights a potential link between type 2 diabetes and altered lipid metabolism in the nervous system. SCD1 activation in conditions of hyperglycemia or insulin resistance may act as a cellular protective mechanism against SFA-induced apoptosis [25]. This mechanism may convert palmitic and stearic acids to palmitoleic and oleic acids, which we found elevated in both the liver and spinal cord. Obesity-induced SCD1 upregulation is reported in several tissues [26], supporting its role as a key regulator of lipid homeostasis. Increased TAG synthesis and accumulation in lipid droplets (mainly hepatic but also neural) may reduce SFA toxicity. Tissue-specific increases in SCD1 activity between the liver and spinal cord in T2D may suggest differential roles in lipid handling. The observed elevation of SCD1 activity in the spinal cord of ZDF rats represents a novel pilot finding, enabling identification of lipid metabolism alterations in this tissue too. Data in the literature indicate that SCD1 is also considered a potential therapeutic target for neurological disorders [27]. Diabetes mellitus is accompanied by multiple neurological complications, which have a pathobiochemical basis in lipid imbalance. It is therefore necessary to investigate these mechanisms in greater detail not only in the brain but also in the spinal cord. This represents a challenge for future research. Our findings of elevated MUFA levels and increased SCD1 activity in the liver are consistent with metabolic alterations also observed in MAFLD, which is frequently comorbid with T2D and characterized by enhanced de novo lipogenesis and hepatic triglyceride accumulation, driven in part by SCD1 upregulation [28].

In contrast to the increase in MUFAs, we observed a significant decrease in the percentage of PUFAs in the liver and also in the spinal cord. The decline in PUFA levels in T2D tissues could adversely affect membrane fluidity, signaling pathways, and the balance of pro-inflammatory and anti-inflammatory mediators. Arachidonic acid (AA) level was significantly reduced in the liver and spinal cord, linked to decreased activity of the enzymes involved in its synthesis from linoleic acid (LA). FADS1 activity in the liver decreased more than threefold; a non-significant increase was observed in the spinal cord. These findings in the liver are consistent with studies reporting reduced serum n-3 and n-6 FA in T2D and reduced FADS1 activity [15] or decreased serum AA and FADS1 activity in newly diagnosed T2D patients [29]. Conversely, Imamura et al. [30] observed higher AA in long-term diabetic patients (median 11 years). In the spinal cord of T2D rats, the reduction in AA (C20:4 n-6) and DGLA (C20:3 n-6) and increase in LA (C18:2 n-6) suggest that ELOVL and FADS2, rather than FADS1, activities are more affected. The reduced C20:4/C18:2 ratio in spinal cord tissue may reflect decreased AA synthesis in the CNS. A significant decrease in free AA in the spinal cord under experimental T2D conditions does not preclude PUFA utilization for the synthesis of prostaglandins and leukotrienes, whose levels, however, we did not measure. These mediators may contribute to the mechanisms through which diabetes could affect processes in the nervous tissue [31]. AA corresponds to approximately 20% of the total neuronal fatty acids and is mainly esterified in membrane phospholipids. Its reduced incorporation into neuronal membranes could compromise membrane integrity and function, thereby contributing to neurodegeneration and neuropathy.

High FADS2 activity (Δ6-desaturase) and low FADS1 (Δ5-desaturase) activity have been associated with insulin resistance and, most importantly, have been shown to predict the development of diabetes [32,33,34]. Our results in the liver are in agreement with the literature [35]. On the other hand, the effect of T2D on FADS1 and FADS2 activities was not confirmed in the spinal cord.

### Limitations and Future Directions

Our study has several limitations, including the relatively small number of experimental animals and the exclusive focus on the spinal cord without examination of other CNS regions. Moreover, histological, immunological, and molecular analyses were not performed, which would provide additional mechanistic insights into the observed alterations in fatty acid metabolism. In addition, desaturase “activities” were inferred from product-to-precursor ratios, which represent indirect estimates influenced by multiple pathways; direct measurements of enzyme expression or activity would be necessary to confirm these results.

This work should be considered a pilot study that provides initial evidence and highlights the need for further research. Future investigations should therefore expand to include other parameters, assess enzyme expression, evaluate pro-inflammatory eicosanoids, and integrate histological and molecular approaches.

## 5. Conclusions

The study results indicate that type 2 diabetes triggers tissue-specific alterations in desaturase activities. Significant changes in MUFA and PUFA content were identified in both the spinal cord and liver of rats with spontaneous T2D. The observed elevation of SCD1 activity in the liver and spinal cord appears to represent an adaptive mechanism to SFA accumulation within these tissues. The decreased content of free PUFAs seems to be a consequence of changes in the activity of the desaturase and elongation system associated with type 2 diabetes. In contrast to the liver, the spinal cord of T2D rats exhibited a reduction in AA (C20:4 n-6) and DGLA (C20:3 n-6), paralleled by an increased representation of LA (C18:2 n-6). Based on these findings, we hypothesize that in the spinal cord, ELOVL and FADS2, rather than FADS1, activities might be predominantly affected.

## Figures and Tables

**Table 1:** Concentrations of plasma insulin, whole-blood glucose, and body weight at 36 weeks of age in rats.

	Glucose (mmol/L)	Insulin (ng/mL)	Body Weight (g)
CONT (n = 15)	6.9 ± 0.67	3.24 ± 0.37	423.5 ± 38
T2D (n = 8)	24.5 ± 2.01 **^●^**	11.52 ± 2.3 **^●^**	528 ± 47.1 **^●^**

^●^ *p* < 0.05 significance between T2D and control group. Abbreviations: CONT—control group (ZDF fa/+); T2D—hyperglycemic ZDF fa/fa group.

**Table 2:** Content of saturated and unsaturated fatty acids in the liver and spinal cord.

		MUFAs (%)	PUFAs (%)	SFAs (%)	n-3/n-6
Liver (n = 23)	CONT (n = 15)	19.5 ± 1.1	40.7 ± 1.2	39.79 ± 1.03	0.17
	T2D (n = 8)	32.6 ± 6.8 **^●^**	30.8 ± 4.8 **^●^**	30.5 ± 1.3	0.21 **^●^**
Spinal cord (n = 23)	CONT (n = 15)	38.4 ± 1.46	31.42 ± 3.1	28.9 ± 4.11	0.34
	T2D (n = 8)	44.8 ± 3.9 **^●^**	27.96 ± 4.1 **^●^**	27.1 ± 2.6	0.25

^●^ *p* < 0.05 significance between T2D and control group. Abbreviations: MUFAs—monounsaturated fatty acids; PUFAs—polyunsaturated fatty acids; SFAs—saturated fatty acids; CONT—control group (ZDF fa/+); T2D—hyperglycemic ZDF fa/fa group.

**Table 3:** Activities of desaturation enzymes.

	Spinal Cord		Liver	
	CONT	T2D	CONT	T2D
C16:1/C16:0 (SCD1)	0.07	0.2	0.07	0.13
C18:1/C18:0 (SCD1)	1.3	2.2	0.86	2.8
C20:4/C18:2 (FADS2 + ELOVL + FADS1)	3.68	2.24	1.25	0.7
C18:3/C18:2 (FADS2)	-	-	0.015	0.04
C20:4/C20:3 (FADS1)	15.5	20.8	26.6	8.1

Abbreviations: CONT—control group (ZDF fa/+); T2D—hyperglycemic ZDF fa/fa group; SCD1—Δ9-desaturase FADS1—fatty acid desaturase-1 (Δ5-desaturase); FADS2—fatty acid desaturase-2 (Δ6-desaturase); ELOVL—elongase of very long-chain fatty acids.

## Data Availability

The data presented in this study are available on request from the corresponding author.

## References

[1] Marrano N., Biondi G., Borrelli A., Rella M., Zambetta T., Di Gioia L., Caporusso M., Logroscino G., Perrini S., Giorgino F. (2023). Type 2 diabetes and Alzheimer’s disease: The emerging role of cellular lipotoxicity. Biomolecules.

[2] Biondi G., Marrano N., Borrelli A., Rella M., Palma G., Calderoni I., Siciliano E., Lops P., Giorgino F., Natalicchio A. (2022). Adipose Tissue Secretion Pattern Influences β-Cell Wellness in the Transition from Obesity to Type 2 Diabetes. Int. J. Mol. Sci..

[3] Petersen M.C., Shulman G.I. (2017). Roles of diacylglycerols and ceramides in hepatic insulin resistance. Trends Pharmacol. Sci..

[4] Sun Q., Xing X., Wang H., Wan K., Fan R., Liu C., Wang Y., Wu W., Wang Y., Wang R. (2024). SCD1 is the critical signaling hub to mediate metabolic diseases: Mechanism and the development of its inhibitors. Biomed. Pharmacother..

[5] Liu X., Strable M.S., Ntambi J.M. (2011). Stearoyl CoA desaturase 1: Role in cellular inflammation and stress. Adv. Nutr..

[6] Pedersen J. (2025). Monounsaturated Fatty Acids in Cardiovascular Disease. Nutrients.

[7] Heeren J., Scheja L. (2021). Metabolic-associated fatty liver disease and lipoprotein metabolism. Mol. Metab..

[8] Wang B., Wu L., Chen J., Dong L., Chen C., Wen Z., Hu J., Fleming I., Wang D.W. (2021). Metabolism pathways of arachidonic acids: Mechanisms and potential therapeutic targets. Signal Transduct. Target. Ther..

[9] Huang C.C., Chang M.T., Leu H.B., Yin W.H., Tseng W.K., Wu Y.W., Lin T.-H., Yeh H.-I., Chang K.-C., Wang J.H. (2020). Association of arachidonic acid-derived lipid mediators with subsequent onset of acute myocardial infarction in patients with coronary artery disease. Sci. Rep..

[10] Aradhyula V., Reddy V.S., Manne M., Siva A.B., Reddy P.P. (2024). Transcriptomic analysis of arachidonic acid pathway across comorbid diseases. Genes.

[11] Rezende B., Alencar A.K.N., de Bem G.F.D., Fontes-Dantas F.L., Montes G.C. (2023). Endocannabinoid System: Chemical Characteristics and Biological Activity. Pharmaceuticals.

[12] Salem A., Kim S.H., Preedy V.R. (2023). Arachidonic acid. Essential Fatty Acids: Sources, Processing Effects, and Health Benefits.

[13] Lowe H., Toyang N., Steele B., Bryant J., Ngwa W. (2021). The endocannabinoid system: A potential target for the treatment of various diseases. Int. J. Mol. Sci..

[14] Jarullah H.H., Saleh E.S. (2025). Influence of Fatty Acid Desaturase Enzyme-1 Gene (FADS1) Gene (rs174547) polymorphism on serum polyunsaturated fatty acids and desaturase activity in type 2 diabetes patients. Int. J. Mol. Sci..

[15] Al A., Kupai K., Veszelka M., Szűcs G., Attieh Z.K., Murlasits Z., Török S., Pósa A., Varga C. (2016). Experimental diabetes mellitus in different animal models. J. Diabetes Res..

[16] Pandey S., Dvorakova M.C. (2020). Future perspective of diabetic animal models. Endocr. Metab. Immune Disord.-Drug Targets.

[17] Kollarova M., Chomova M., Radosinska D., Tothova L., Shawkatova I., Radosinska J. (2022). ZDF (fa/fa) rats show increasing heterogeneity in main parameters during ageing, as confirmed by biometrics, oxidative stress markers and MMP activity. Exp. Physiol..

[18] Hamano F. (2020). Quantification of Fatty Acids in Mammalian Tissues by Gas Chromatographic Methods. J. Lipid Res..

[19] Vessby B., Gustafsson I.-B., Tengblad S., Boberg M., Andersson A. (2002). Desaturation and elongation of fatty acids and insulin action. Ann. N. Y. Acad. Sci..

[20] Ntambi J.M., Buhrow S.A., Kaestner K.H., Christy R.J., Sibley E., Kelly T.J., Lane M.D. (1988). Differentiation-induced gene expression in 3T3-L1 preadipocytes: Characterization of a differentially expressed gene encoding stearoyl-CoA desaturase. J. Biol. Chem..

[21] McMillan D.W., Bigford G.E., Farkas G.J. (2023). The physiology of neurogenic obesity: Lessons from spinal cord injury research. Obes. Facts.

[22] Miyake T., Furukawa S., Matsuura B., Yoshida O., Miyazaki M., Shiomi A., Kanzaki S., Nakaguchi H., Sunago K., Nakamura Y. (2022). Plasma Fatty Acid Composition Is Associated with Histological Findings of Nonalcoholic Steatohepatitis. Biomedicines.

[23] Paton C.M., Ntambi J.M. (2009). Biochemical and physiological function of stearoyl-CoA desaturase. Am. J. Physiol. Endocrinol. Metab..

[24] Luiken J.J.F.P., Arumugam Y., Dyck D.J., Bell R.C., Pelsers M.M., Turcotte L.P., Tandon N.N., Glatz J.F.C., Bonen A. (2001). Increased rates of fatty acid uptake and plasmalemmal fatty acid transporters in obese Zucker rats. J. Biol. Chem..

[25] Ricchi M., Odoardi M.R., Carulli L., Anzivino C., Ballestri S., Pinetti A., Fantoni L.I., Marra F., Bertolotti M., Banni S. (2009). Differential effect of oleic and palmitic acid on lipid accumulation and apoptosis in cultured hepatocytes. J. Gastroenterol. Hepatol..

[26] Hulver M.W., Berggren J.R., Carper M.J., Miyazaki M., Ntambi J.M. (2005). Elevated stearoyl-CoA desaturase-1 expression in skeletal muscle contributes to abnormal fatty acid partitioning in obese humans. Cell Metab..

[27] Loix M., Vanherle S., Turri M., Kemp S., Fernandes K.J., Hendriks J.J., Bogie J.F. (2024). Stearoyl-CoA desaturase-1: A potential therapeutic target for neurological disorders. Mol. Neurodegener..

[28] Jeyakumar S.M., Vajreswari A. (2022). Stearoyl-CoA desaturase 1: A potential target for non-alcoholic fatty liver disease?—perspective on emerging experimental evidence. World J. Hepatol..

[29] van Woudenbergh G.J., Kuijsten A., van der Kallen C.J., van Greevenbroek M.M., Stehouwer C.D., Blaak E.E., Feskens E.J.M. (2012). Comparison of fatty acid proportions in serum cholesteryl esters among people with different glucose tolerance status: The CoDAM study. Nutr. Metab. Cardiovasc. Dis..

[30] Imamura F., Micha R., Wu J.H., de Oliveira Otto M.C., Otite F.O., Abete Y., Ding I., Koulman S.L., Hu J., Sakurai S. (2014). Plasma polyunsaturated fatty acid profile and delta-5 desaturase activity are altered in patients with type 2 diabetes. Metabolism.

[31] Thomas M.H., Pelleïeux S., Vitale N., Olivier J.L. (2016). Arachidonic acid in Alzheimer’s disease. J. Neurol. Neuromed..

[32] Peña-Bautista C., Vento M., Baquero M., Cháfer-Pericás C. (2019). Lipid peroxidation in neurodegeneration. Clin Chim Acta..

[33] Patel P.S., Sharp S.J., Jansen E., Luben R.N., Khaw K.T., Wareham N.J., Forouhi N.G. (2010). Fatty acids measured in plasma and erythrocyte-membrane phospholipids and derived by food-frequency questionnaire and the risk of new-onset type 2 diabetes: A pilot study in the EPIC-Norfolk cohort. Am. J. Clin. Nutr..

[34] Kröger J., Zietemann V., Enzenbach C., Weikert C., Jansen E.H.J., Döring F., Joost H.-G., Boeing H., Schulze M.B. (2011). Erythrocyte membrane phospholipid fatty acids, desaturase activity, and dietary fatty acids in relation to risk of type 2 diabetes in the EPIC-Potsdam Study. Am. J. Clin. Nutr..

[35] Tosi F., Sartori F., Guarini P., Olivieri O., Martinelli N., Camps J. (2014). Delta-5 and delta-6 desaturases: Crucial enzymes in polyunsaturated fatty acid-related pathways with pleiotropic influences in health and disease. Oxidative Stress and Inflammation in Non-Communicable Diseases—Molecular Mechanisms and Perspectives in Therapeutics.

